# Enhancing therapeutic efficacy of human adipose-derived stem cells by modulating photoreceptor expression for advanced wound healing

**DOI:** 10.1186/s13287-022-02892-2

**Published:** 2022-05-26

**Authors:** Sang Ho Lee, Yu-Jin Kim, Yeong Hwan Kim, Han Young Kim, Suk Ho Bhang

**Affiliations:** 1 School of Chemical Engineering, Sungkyunkwan University, Suwon, 16419 Republic of Korea; 2 Department of Biomedical-Chemical Engineering, The Catholic University of Korea, Bucheon, 14662 Republic of Korea

**Keywords:** Human adipose-derived stem cells, Organic light-emitting diodes, Photobiomodulation, Opsins, Wound healing

## Abstract

**Background:**

Human adipose-derived stem cells (hADSCs) have been widely used for regenerative medicine because of their therapeutic efficacy and differentiation capacity. However, there are still limitations to use them intactly due to some difficulties such as poor cell engraftment and viability after cell transplantation. Therefore, techniques such as photobiomodulation (PBM) are required to overcome these limitations. This study probed improved preclinical efficacy of irradiated hADSCs and its underlying molecular mechanism.

**Methods:**

hADSCs were irradiated with green organic light-emitting diodes (OLEDs). Treated cells were analyzed for mechanism identification and tissue regeneration ability verification. Expression levels of genes and proteins associated with photoreceptor, cell proliferation, migration, adhesion, and wound healing were evaluated by performing multiple assays and immunostaining. Excision wound models were employed to test in vivo therapeutic effects.

**Results:**

In vitro assessments showed that Opsin3 (OPN3) and OPN4 are both expressed in hADSCs. However, only OPN4 was stimulated by green OLED irradiation. Cell proliferation, migration, adhesion, and growth factor expression in treated hADSCs were enhanced compared to control group. Conditioned medium containing paracrine factors secreted from irradiated hADSCs increased proliferation of human dermal fibroblasts and normal human epidermal keratinocytes. Irradiated hADSCs exerted better wound healing efficacy in vivo than hADSCs without OLED irradiation.

**Conclusions:**

Our study introduces an intracellular mechanism of PBM in hADSCs. Our results revealed that photoreceptor OPN4 known to activate G_q_-protein and consequently lead to reactive oxygen species production responded to OLED irradiation with a wavelength peak of 532 nm. In conclusion, green OLED irradiation can promote wound healing capability of hADSCs, suggesting that green OLED has potential preclinical applications.

## Background

Human adipose-derived stem cells (hADSCs) have galvanized researchers working in the field of regenerative medicine due to their unique biological characteristics and promising preclinical potential. In the past few decades, hADSCs have been increasingly revealed to hold potential as innate biotherapeutics. There is compelling evidence supporting the possibility of using hADSCs for promoting tissue regeneration [1–3]. Although several studies have explored the innate therapeutic potency of hADSCs for various diseases and their modes of action [4], there still exist several drawbacks of hADSCs such as low survival rate, heterogeneity, and inconsistent therapeutic outcomes [5–7]. Therefore, many recent studies have used various techniques to improve the therapeutic efficacy of hADSCs [8, 9]. One strategy to augment the therapeutic potential of hADSCs is to perform photobiomodulation (PBM). PBM has been widely used to treat diseases by introducing a light source such as a laser or a light-emitting diode (LED) to target cells or tissues [10, 11]. Growing evidence suggests that PBM may lead to physical and chemical effects in target cells by modulating various biological processes such as cell proliferation and inflammatory responses [12, 13]. Furthermore, PBM can be applied to achieve enhanced expression of desired biomolecules that contribute to regeneration of injured tissues.

Previous studies have elucidated that cellular changes caused by PBM are due to production of adenosine triphosphate (ATP), which is related to mitochondrial cytochrome c oxidase (CCO) [14, 15]. Electron transfer through CCO can generate a proton gradient that provides energy to ATP synthase for catalysis of ATP production [16]. Importantly, CCO is known to increase reactive oxygen species (ROS) levels in treated cells [17]. Suh et al. have demonstrated that PBM can mediate cell signaling pathways related to expression of opsins (OPNs) known to be G-protein-coupled receptors (GPCRs) [18, 19]. Activating transient receptor potential (TRP) channels is one of action mechanisms of OPNs [20]. When TRP channels are activated, they allow calcium ions (Ca^2+^) to flow into the inner membrane, thereby raising intracellular Ca^2+^ concentration [21]. Other signaling pathways of OPNs involve G_q_-protein [20]. G_q_ activation can induce Ca^2+^ release from the endoplasmic reticulum (ER) into the cytoplasm of cells [22]. As reported by Gordeeva et al. [23], intracellular Ca^2+^ level elevation can trigger ROS generation. Therefore, it is important to determine the proper mechanism associated with light source used for PBM.

In previous studies of PBM, experiments were conducted by short exposure of target cells to high-intensity light using lasers or LEDs as power sources [10, 24]. However, excessive intensities of those lights may generate heat that may serve as additional exogenous stimuli. Thus, a big question when using lasers or LEDs as light sources for PBM is whether alterations of biological processes and changes in phenotypic profiles of exposed cells are caused by irradiation or heat generated from light sources [25, 26]. Because there are contrasting results when cells are irradiated with light of similar wavelengths, the application of PBM remains controversial, due to the lack of investigations regarding the correlation between wavelengths and corresponding cellular responses [27, 28]. In previous experiments using PBM as exogenous stimuli for hADSCs, researchers have focused on applying PBM particularly for modulating differentiation of cells [29, 30]. PBM has also been widely adopted to study differences in apoptosis, cell proliferation, migration, and paracrine factor secretion of hADSCs with different wavelength bands [31]. Based on recent studies reporting that PBM encourages the secretion of paracrine factors from stem cells [32], therapeutic effects of conditioned medium (CM) derived from hADSCs exposed to blue, green, and red light are now being actively studied.

**Fig. 1:**
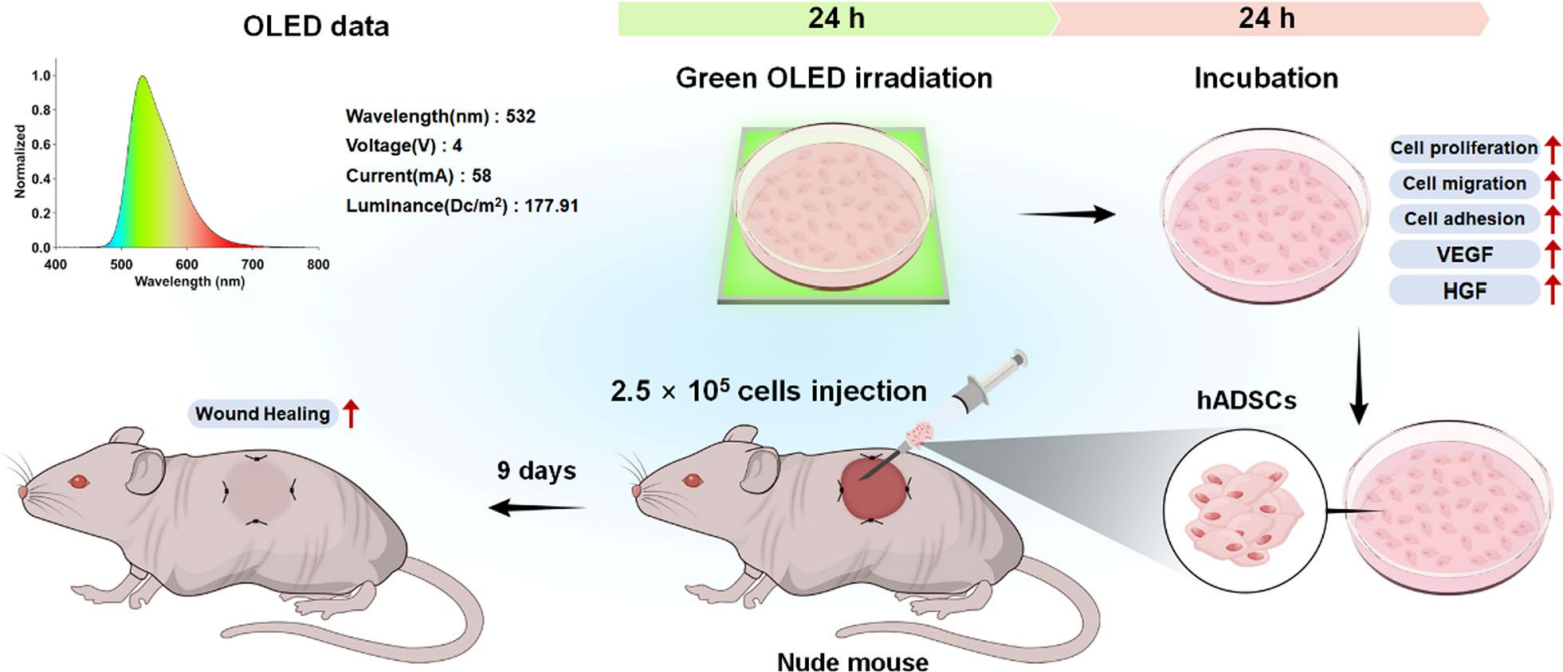
Schematics of experimental design using green OLED and hADSCs

In the present study, we used organic LEDs (OLEDs) with a low heat generation and relatively weak intensity for PBM to evaluate cellular activities in response to a specific wavelength. OLED applied in our experiment has been commercialized with officially confirmed uniform properties. It has not been applied to stem cell-related research previously. Based on previous studies showing that responses in cells irradiated with green light can improve wound healing without obvious cytotoxicity [33, 34], herein we utilized green light with a wavelength peak of 532 nm. Due to the lack of studies investigating the expression of OPNs in hADSCs in response to PBM, we explored cellular mechanisms involved in the effects of PBM by determining the expression of OPNs. Furthermore, we determined effects of OLED-based PBM on secretion of growth factors, proliferation, migration, and adhesion of hADSCs, all of which are known to play important roles in cutaneous wound healing [35, 36]. Subsequently, we optimized irradiation conditions of PBM for hADSCs. Such conditions were used to treat hADSCs which were then applied to mouse cutaneous wound models to evaluate their therapeutic effects in vivo (Fig. 1).

## Methods

### Cell culture

hADSCs and human dermal fibroblasts (hDFs) were purchased from Lonza (Basel, Switzerland) and cultured in Dulbecco’s modified Eagle’s medium (Gibco BRL, Gaithersburg, MD, USA) supplemented with 10% (*v*/*v*) fetal bovine serum (Gibco BRL) and 1% (*v*/*v*) penicillin/streptomycin (Gibco BRL). Normal human epidermal keratinocytes (NHEKs) were purchased from PromoCell (Heidelberg, Germany) and cultured in keratinocyte growth medium 2 (PromoCell) supplemented with 1% (*v*/*v*) penicillin/streptomycin. These cells were incubated at 37 ℃ in an atmosphere of 5% CO_2._ Culture media were changed every 2 days.

### Green OLED irradiation

An OLED panel was purchased from Kaneka (Osaka, Japan). This OLED panel was connected to a regulated direct current power supply with the voltage set at 4 V. hADSCs-seeded tissue culture dishes and plates were placed on the OLED panel inside an incubator. After washing with phosphate-buffered saline (PBS, Gibco BRL) followed by a medium exchange, cells were irradiated with a wavelength peak of 532 nm for an optimized period.

### Quantitative real-time polymerase chain reaction

Relative gene expression levels of human *GAPDH*, *OPN3*, *OPN4*, *PLC-β, IP3R, C-MYC*, *CXCL12*, *CXCR4*, *β-CATENIN*, *INTEGRIN α5*, *INTEGRIN β1, VEGF*, and *HGF* in green OLED-treated hADSCs, human *GAPDH*, *KI67*, *PCNA*, and *C-MYC* in conditioned media-treated hDFs and NHEKs, and mouse *β-Actin*, *Acta2*, *Cd31*, and *Laminin* in in vivo wound regions were quantified by quantitative real-time polymerase chain reaction (qRT-PCR). The sequences of primers used for qRT-PCR are listed in Table 1. hADSCs, hDFs, and NHEKs were seeded into 6-well tissue culture plates at a density of 1 × 10^5^ cells/well. Ribonucleic acids (RNAs) were then extracted from cells and in vivo samples using 1 mL of TRIzol (Ambion, Austin, TX, USA) and 200 μL of chloroform (Sigma-Aldrich, St. Louis, MO, USA). RNA pellets were obtained by centrifugation at 12,000 rpm for 10 min at 4 °C. After dissolving the pellets in ribonuclease-free dH_2_O (TaKaRa, Kusatsu, Japan) and PrimeScript RT Master Mix (TaKaRa), PCR amplification was performed using synthesized complementary DNA as template. iQ SYBR green supermix (Bio-Rad, Hercules, CA, USA) and CFX connect RT-PCR detection system (Bio-Rad) were used for qRT-PCR analysis.

**Table 1:** Quantitative real-time polymerase chain reaction (qRT-PCR) primer sequences

Gene	Primer	Sequence (5′–3′)
*Human GAPDH*	Forward	GTCGGAGTCAACGGATTTGG
	Reverse	GGGTGGAATCAATTGGAACAT
*Human OPN3*	Forward	CATTGCTATGGCCATATTCTATATTCCATTCG
	Reverse	ATCATTAAAAAGCACATTTTGGCCAGTTTC
*Human OPN4*	Forward	GTTGCTGACATCCTGCTCCT
	Reverse	TCCCGGATGGCCCTGAAGAT
*Human PLC-β*	Forward	CTGGATGAAAAGCCCAAGCTG
	Reverse	ATTGCTGTCTTCACTGATCTTTCCT
*Human IP3R*	Forward	GGTTGGAGACTATCAGCTCGCT
	Reverse	GCATCATTGGGCTGAACTGGTG
*Human C-MYC*	Forward	AATGAAAAGGCCCCCAAGGTAGTTATCC
	Reverse	GTCGTTTCCGCAACAAGTCCTCTTC
*Human CXCL12*	Forward	TGCCAGAGCCAACGTCAAG
	Reverse	CAGCCGGGCTACAATCTGAA
*Human CXCR4*	Forward	TGCTTGCTGAATTGGAAGTG
	Reverse	AGTCATAGTCCCCTGAGCCC
*Human β-CATENIN*	Forward	TTGTGCGGCGCCATTTTAAG
	Reverse	TCCTCAGACCTTCCTCCGTC
*Human INTEGRIN α5*	Forward	TGCAGTGTGAGGCTGTGTACA
	Reverse	GTGGCCACCTGACGCTCT
*Human INTEGRIN β1*	Forward	GCCGCGCGGAAAAGATGAAT
	Reverse	CACAATTTGGCCCTGCTTGTA
*Human VEGF*	Forward	GAGGGCAGAATCATCACGAAGT
	Reverse	CACCAGGGTCTCGATTGGAT
*Human HGF*	Forward	GATGGCCAGCCGAGGC
	Reverse	TCAGCCCATGTTTTAATTGCA
*Human KI67*	Forward	TGACCCTGATGAGAAAGCTCAA
	Reverse	CCCTGAGCAACACTGTCTTTT
*Human PCNA*	Forward	CCTGCTGGGATATTAGCTCCA
	Reverse	CAGCGGTAGGTGTCGAAGC
*Mouse β-Actin*	Forward	GGCTGTATTCCCCTCCATCG
	Reverse	CCAGTTGGTAACAATGCCATGT
*Mouse Acta2*	Forward	CAGGCATGGATGGCATCAATCAC
	Reverse	ACTCTAGCTGTGAAGTCAGTGTCG
*Mouse Cd31*	Forward	CAAACAGAAACCCGTGGAGATG
	Reverse	ACCGTAATGGCTGTTGGCTTC
*Mouse Laminin*	Forward	TCCTGCCCACATCAAACAGA
	Reverse	GTAGGTCAAAGGCTCGGCTC

### Calcium assay

Intracellular calcium concentration in green OLED-treated hADSCs was measured using a calcium detection assay kit (ab102505; Abcam, Cambridge, UK). In this study, 1 × 10^6^ of hADSCs were treated with green OLED and lysed with calcium assay buffer. After centrifugation at 27,000×*g* for 5 min at 4 °C, supernatants were collected. Samples were mixed with chromogenic reagent and incubated at room temperature for 10 min. Optical density of each sample was then measured at 575 nm using a microplate reader (Infinite F50; Tecan, Männedorf, Switzerland).

### Western Blot

Expression levels of various proteins in green OLED-treated hADSCs were detected by Western Blotting. In this study, 1 × 10^6^ of hADSCs were treated with green OLED and lysed with radio-immunoprecipitation assay lysis buffer (Rockland Immunochemicals Inc., Limerick, PA, USA) for each sample. After centrifugation at 8000×*g* for 10 min at 4 °C, supernatants were collected as protein extracts. Pierce BCA protein assay kit (Pierce Biotechnology, Rockford, IL, USA) was used to determine protein concentration, following the manufacturer’s protocol. Samples with equal amounts of proteins were denatured in Pierce LDS sample buffer (Pierce Biotechnology) at 100 °C for 5 min. After separating proteins by sodium dodecyl sulfate–polyacrylamide gel electrophoresis, fractionated proteins were transferred onto Immun-blot PVDF membranes (Bio-Rad). Membranes were then blocked with 5% (*w*/*v*) skim milk in Tris-buffered saline (TBS, Bio-Rad) with 0.05% (*v*/*v*) Tween 20 (Bio-Rad) (TBST) and incubated with anti-GAPDH (AF5718; R&D Systems, Minneapolis, MN, USA), anti-OPN3 (ab228748; Abcam), anti-OPN4 (LS-C384509; LSBio, Seattle, WA, USA), anti-BAX (#5023; Cell signaling, Danvers, MA, USA), anti-Bcl-2 (ab182858; Abcam), and anti-KI67 (ab16667; Abcam) antibodies overnight at 4 °C. After washing with TBST, membranes were incubated with horseradish peroxidase conjugated antibodies (HAF017 for GAPDH, and HAF008 for OPN3, OPN4, BAX, Bcl-2, and KI67, R&D Systems) at room temperature for 1 h. After washing membranes with TBST, enhanced chemiluminescence solution (TransLab, Daejeon, Korea) was added to membranes. Blots were then developed using X-ray films (AGFA CP-BU NEW; Agfa HealthCare NV, Mortsel, Belgium).

### Cellular ROS and ATP assay

Relative levels of ROS in green OLED-treated hADSCs were evaluated by cellular ROS assay using 2′,7′-dichlorodihydrofluorescein diacetate (H2DCFDA, Invitrogen, Carlsbad, CA, USA). hADSCs for cellular ROS assay were seeded into 96-well tissue culture plates at a density of 3 × 10^3^ cells/well. After cells were incubated in 10 μM H2DCFDA at 37 °C for 20 min, the fluorescence intensity (Ex/Em = 494 nm/524 nm) was measured using a microplate reader (Varioskan LUX multimode microplate reader; Thermo Fisher Scientific, Waltham, MA, USA). hADSCs for DCFDA staining were seeded into 6-well tissue culture plates at a density of 7 × 10^4^ cells/well. After these cells were incubated in 10 μM H2DCFDA at 37 °C for 2 min, they were examined using a fluorescence microscope (DFC 3000 G; Leica Microsystems GmbH, Wetzlar, Germany). ATP assay kit (ab83355; Abcam) was used for evaluating the amount of ATP in green OLED-treated hADSCs, following the manufacturer’s protocol. Each cell lysate sample was prepared from 1 × 10^6^ of hADSCs.

### Terminal deoxynucleotidyl transferase dUTP nick end labeling (TUNEL) staining

Cytotoxicity of green OLED irradiation was analyzed by TUNEL staining using an ApopTag fluorescein in situ apoptosis detection kit (Merck KGaA, Darmstadt, Germany). Briefly, hADSCs were seeded into 6-well tissue culture plates at a density of 7 × 10^4^ cells/well. After green OLED irradiation, cells were fixed with 4% paraformaldehyde (Biosesang, Seongnam, Korea). Equilibration buffer, working strength TdT enzyme, stop/wash buffer, and working strength anti-digoxigenin conjugate were applied to fixed cells followed by PBS washing after each step. For counterstaining, 4',6-diamidino-2-phenylindole (DAPI, Vector Laboratories, Burlingame, CA, USA) was used. Hydrogen peroxide solution (Sigma-Aldrich) was used for a positive control of TUNEL analysis [37]. Stained cells were examined using a fluorescence microscope (DFC 3000 G).

### Fluorescein diacetate (FDA) and ethidium bromide (EB) staining

FDA/EB staining was performed to assess cytotoxicity of green OLED irradiation. hADSCs were seeded into 6-well tissue culture plates at a density of 7 × 10^4^ cells/well. The staining solution was prepared using 10 mL of FDA (Sigma-Aldrich) stock solution (1.5 mg/mL of FDA in dimethyl sulfoxide), 5 mL of EB (Sigma-Aldrich) stock solution (1 mg/mL of EB in PBS), and 3 mL of PBS. Green OLED-treated cells were washed with PBS and incubated in the staining solution at 37 °C for 2 min. After washing with PBS, stained cells were examined using a fluorescence microscope (DFC 3000 G).

### Immunocytochemistry

hADSCs were seeded into 6-well tissue culture plates at a density of 5 × 10^4^ cells/well. After green OLED irradiation, cells were fixed with 4% paraformaldehyde. These fixed cells were blocked with 10% (*v*/*v*) normal goat serum (Jackson Immuno Research Laboratories, West Grove, PA, USA) and 0.6% (*v*/*v*) Triton X-100 (Sigma-Aldrich) in PBS at room temperature for 1 h. Cells were then immunofluorescence stained with anti-KI67 antibody (ab16667; Abcam) and fluorescein (FITC)-conjugated AffiniPure goat anti-rabbit IgG (H + L) (Jackson Immuno Research Laboratories). DAPI was used as a counterstain. Stained cells were examined using a fluorescence microscope (DFC 3000 G).

### Cell counting kit-8 (CCK-8) assay

Cell viability was evaluated using CCK-8 assay kit (Dojindo Molecular Technologies, Inc., Kumamoto, Japan). Briefly, hADSCs were seeded into 24-well tissue culture plates at a density of 1.5 × 10^4^ cells/well. Green OLED-treated cells were washed with PBS and incubated with 10% (*v*/*v*) CCK-8 solution in culture medium at 37 °C for 2 h. Optical density of each sample was then measured at 450 nm using a microplate reader (Infinite F50).

### Cell scratch assay

Cell migration was analyzed by cell scratch assay [38]. Briefly, hADSCs were seeded into 24-well tissue culture plates at a density of 1.5 × 10^4^ cells/well. SPLScar scratcher (SPL Life Sciences, Pocheon, Korea) was used to scratch cells prior to green OLED irradiation. Cell-covered area was estimated as follows: [1 − (pixel size of uncovered area at each time point)/(pixel size of initially scratched area)] × 100%[39].

### Enzyme-linked immunosorbent assay (ELISA)

Secretion levels of SDF-1, VEGF, and HGF proteins into medium of green OLED-treated hADSCs were quantified by ELISA. Briefly, hADSCs were seeded into 6-well tissue culture plates at a density of 1 × 10^5^ cells/well. Supernatants were collected and analyzed using ELISA kits (R&D Systems). Diluted capture antibody was added to plates and incubated at room temperature overnight. After rinsing with a washing buffer, plates were blocked with reagent diluent. Samples, diluted detection antibody, streptavidin-HRP, and substrate solution were added to plates and incubated at room temperature. Plates were washed with a washing buffer before each step. After adding stop solution to the plate, optical density of each sample was then measured at 450 nm using a microplate reader (correction 540 nm, Infinite F50).

### Phalloidin staining

Phalloidin staining was performed to assess cell adhesion. hADSCs were seeded into 6-well tissue culture plates at a density of 5 × 10^4^ cells/well. These cells were then fixed with 4% paraformaldehyde after green OLED irradiation. Antifade mounting medium with phalloidin (Vector Laboratories) was used for actin filament (F-actin) staining, and DAPI was used as a counterstain. Stained cells were examined using a fluorescence microscope (DFC 3000 G).

### CM treatment

Effects of paracrine factors secreted from green OLED-treated hADSCs were evaluated by CM treatment. Briefly, hADSCs, hDFs, and NHEKs were seeded in 6-well tissue culture plates at a density of 1 × 10^5^ cells/well. Conditioned media were collected from supernatants of hADSCs at 48 h after green OLED irradiation for 24 h and diluted with serum-free media to reduce influences of fetal bovine serum. Media for culturing hDFs and NHEKs were replaced with 25% and 50% (*v*/*v*) CM. Cells were sampled at 48 h after media exchange.

### Full-thickness skin wound model

Five-week-old female athymic nude mice (BALB/c-nude; OrientBio, Seongnam, Korea) were anesthetized by intraperitoneal injection of 100 mg/kg ketamine (Yuhan, Seoul, Korea) and 10 mg/kg xylazine (Bayer, Leverkusen, Germany). Two 10-mm-diameter full-thickness skin wounds were made on the dorsal side of each mouse. Wounds were then fastened to the underlying tissue with 6–0 silk sutures (AILEE, Busan, Korea). After excision, mice were divided into three groups: no treatment (NT, 100 μL of PBS was injected into each wound) served as control, hADSCs (SC, 2.5 × 10^5^ of hADSCs without green OLED irradiation in 100 μL of PBS were injected into each wound), and green OLED-treated hADSCs (GTC, 2.5 × 10^5^ of hADSCs were harvested at 24 h after green OLED irradiation for 24 h in 100 μL of PBS and injected into each wound) groups. Every wound was covered with a dressing (Tegaderm; 3M Health Care, St. Paul, MN, USA) after injection [40]. All procedures were approved by the Institutional Animal Care and Use Committee of Sungkyunkwan University (approval No. SKKUIACUC2020-06-11-1).

### Assessment of wound healing efficacy

Skin covered areas of wounds were quantified by assessing photographs taken every 3 days. Pixel sizes of areas were adjusted according to the ruler taken with and calculated using Adobe Photoshop CC (Adobe systems, CA, USA). Skin covered areas were estimated as follows: [1 − (a pixel size of the wound at each time point)/(a pixel size of the wound on day 0)] × 100% [41].

### Histological analysis

Tissue samples of wound regions were retrieved on day 9 and fixed in 4% paraformaldehyde. Fixed samples were soaked in 30% (*w*/*v*) sucrose in PBS and then embedded in optimum cutting temperature compound (SciGen Scientific, Chicago, IL, USA). Frozen samples were sliced vertically into 10-μm-thick sections. Thereafter, vertical slices were blocked and incubated with anti-laminin (ab11575; Abcam, 1:200) and anti-involucrin (ab53112; Abcam, 1:100) antibodies at 4 °C overnight. After washing with PBS, sections were incubated with fluorescein (FITC)-conjugated AffiniPure goat anti-rabbit IgG (H + L) (1:50) at room temperature for 1 h. Stained tissues were examined using a fluorescence microscope (DFC 3000 G). In vivo specimens were stained with hematoxylin and eosin (H&E) to identify tissue regeneration and structures. Masson’s trichrome staining was performed to examine tissue fibrosis.

### Statistical analysis

Quantitative data are presented as mean ± standard deviation using GraphPad Prism (GraphPad Software, San Diego, CA, USA). All data were analyzed using Student’s *t* test. Statistical significance was considered at *p* < 0.05.

## Results

### Cellular changes in hADSCs induced by green OLED irradiation

**Fig. 2:**
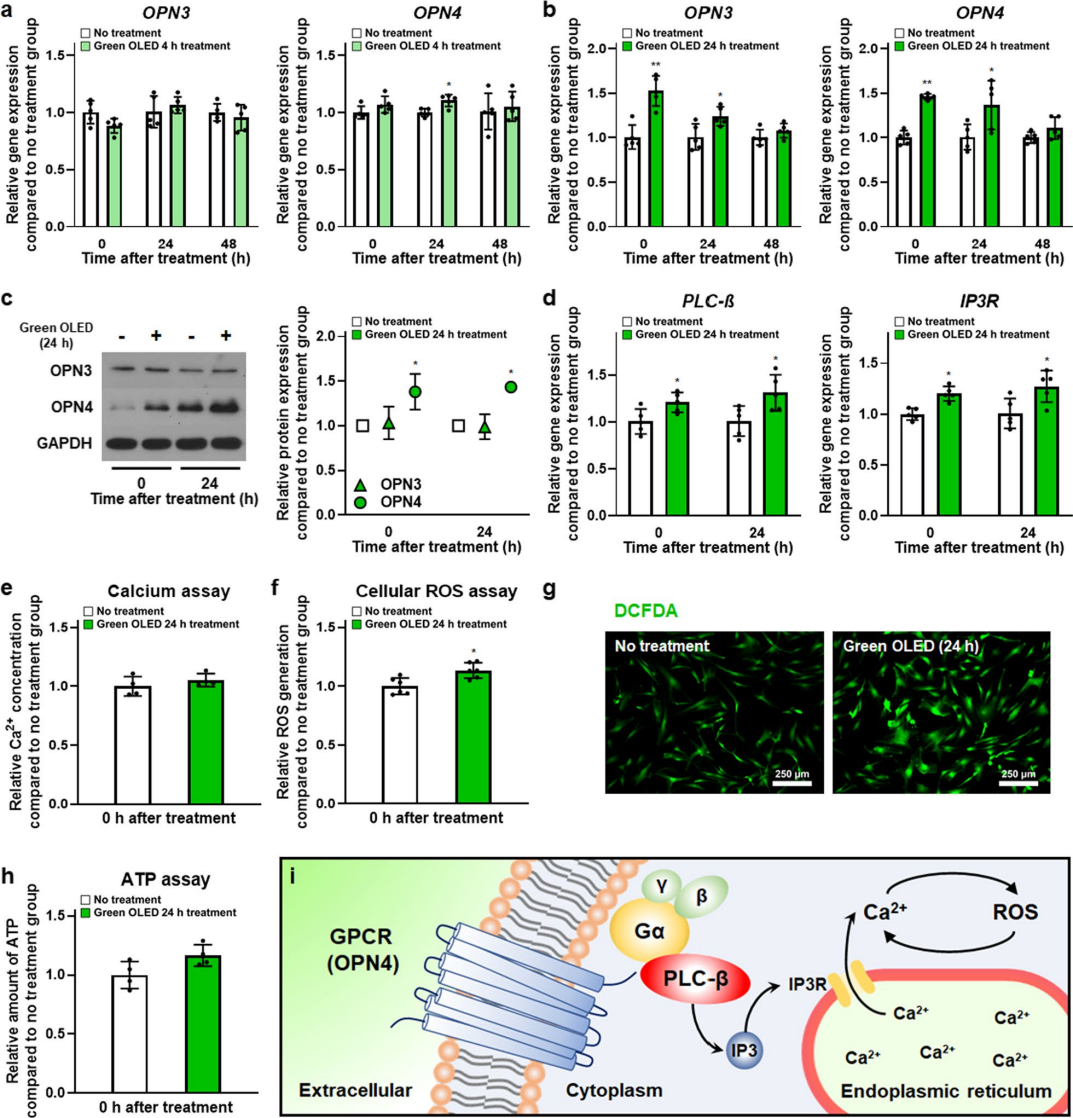
Green OLED irradiation-induced cellular changes in hADSCs. Gene expression levels of *OPN3,* and *OPN4* in hADSCs at 0, 24, and 48 h after green OLED irradiation for 4 h (**a**) or 24 h (**b**) as evaluated by qRT-PCR (*n* = 5). **c** Western Blot analysis of OPN3 and OPN4 expression in hADSCs at 0 and 24 h after green OLED irradiation for 24 h with quantification results (*n* = 3). **d** Gene expression levels of *PLC-β,* and *IP3R* in hADSCs at 0 and 24 h after green OLED irradiation for 24 h as evaluated by qRT-PCR (*n* = 5). **e** Quantification of Ca^2+^ in hADSCs after irradiation for 24 h as evaluated by calcium assay (*n* = 4). **f** Quantification of ROS in hADSCs after irradiation for 24 h as evaluated by cellular ROS assay (*n* = 6). **g** Representative H2DCFDA staining images of hADSCs after green OLED irradiation for 24 h (scale bar = 250 µm). **h** Quantification of ATP in hADSCs after green OLED irradiation for 24 h as evaluated by ATP assay (*n* = 4). **i** Schematic of OPN4 molecular mechanism. *p < 0.05; **p < 0.001 compared to no treatment group with unpaired *t* test

Cellular changes induced by green OLED irradiation in terms of photoreceptor expression, ROS generation, and ATP amount in green OLED-treated hADSCs compared to those in cells without light irradiation were assessed. The opsin family gene expression was evaluated to identify receptors stimulated by green OLED irradiation. As shown in Fig. 2a, b, qRT-PCR results showed significant increases in *OPN3* and *OPN4* gene expression levels after irradiating hADSCs with green OLED for 24 h compared to the NT group. Expression levels of *OPN3* and *OPN4* at 0 and 24 h post-irradiation were significantly elevated in hADSCs irradiated with green OLED for 24 h. In contrast with the group of hADSCs with 24 h of green OLED irradiation, only *OPN4* gene expression was slightly increased in hADSCs irradiated with 4 h green OLED than in hADSCs without light irradiation. To find out the exact photoreceptor stimulated by green OLED irradiation, Western Blot analysis was performed. Only OPN4 protein was found to be upregulated (Fig. 2c). Furthermore, *PLC-β*, and *IP3R* gene expression levels in green OLED-treated hADSCs were analyzed to investigate the mechanism. They were found to be significantly enhanced at 0 and 24 h after irradiation compared to those in the NT group (Fig. 2d).

Amounts of intracellular Ca^2+^, ROS and ATP were evaluated to determine whether molecular behaviors between the green OLED-treated group and the NT group were different. All assays were performed right after irradiation. There was no difference in Ca^2+^ concentration (Fig. 2e), but cellular ROS assay showed a statistically significant difference in intracellular ROS level between the two groups (Fig. 2f). Green OLED-treated hADSCs exhibited higher levels of ROS than untreated cells. Additionally, H2DCFDA staining showed the same tendency (Fig. 2g). This increment might have beneficial effects on treated hADSCs according to other experimental results of this study. However, the amount of ATP in the treated group did not statistically differ from that in the NT group (Fig. 2h). These results indicate that green OLED irradiation stimulates OPN4 expression and consequently enhances intracellular ROS level without ATP production (Fig. 2i).

### Cell viability of hADSCs after green OLED irradiation

**Fig. 3:**
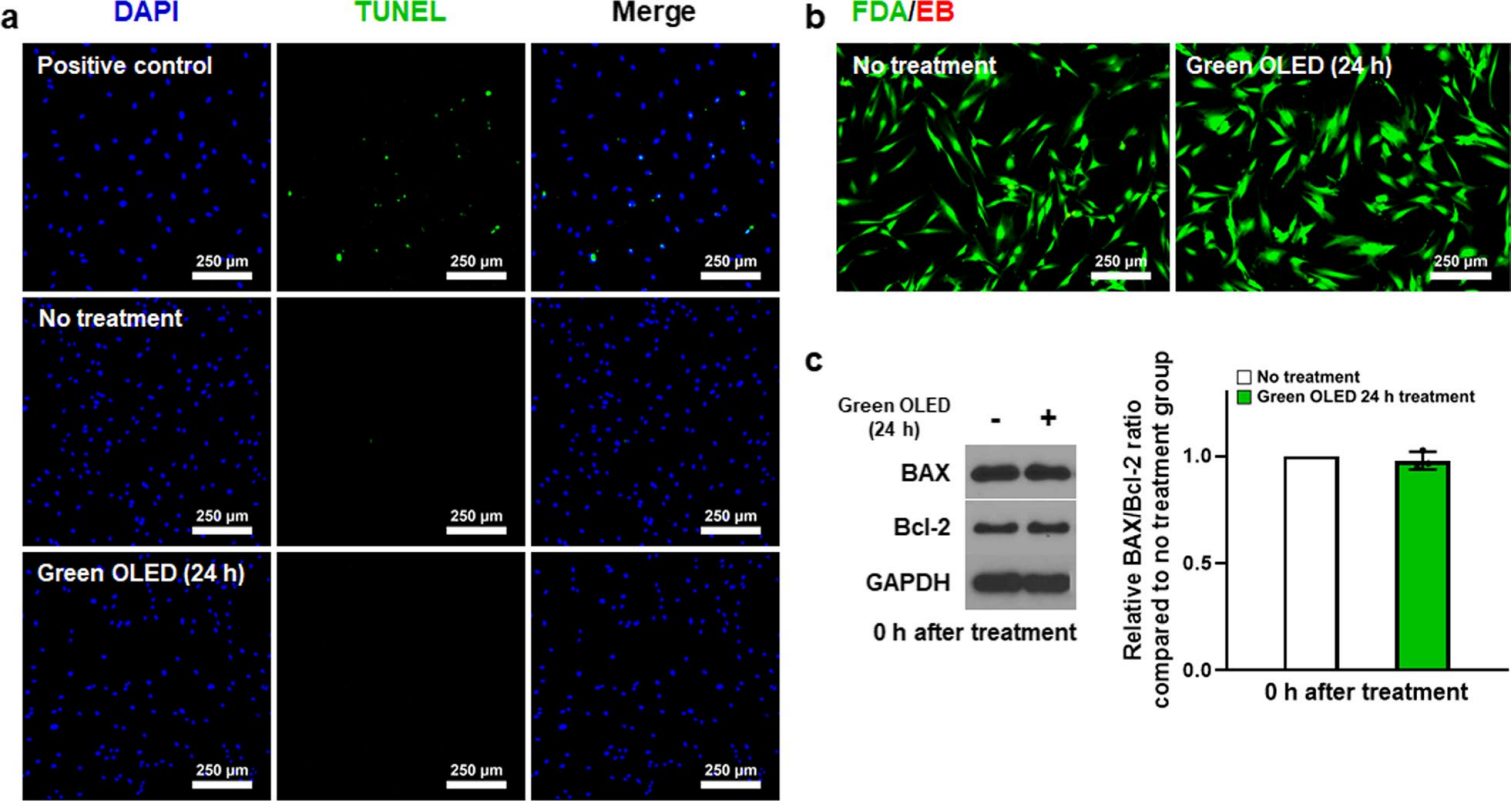
Viability of hADSCs after green OLED irradiation for 24 h. **a** Apoptosis of hADSCs detected by TUNEL staining (blue: nuclei, green: apoptotic cells, scale bar = 250 µm). **b** Cytotoxicity of green OLED irradiation evaluated by FDA/EB staining (green: live cells, red: dead cells, scale bar = 250 µm). **c** Western Blot analysis of BAX and Bcl-2 expression in hADSCs after green OLED irradiation for 24 h and quantification results of BAX/Bcl-2 ratio (*n* = 3)

Apoptotic activities of hADSCs induced by green OLED irradiation were evaluated by TUNEL assay, FDA/EB staining, and Western Blot analysis. As shown in Fig. 3a, b, TUNEL and FDA/EB images did not reveal any apoptotic or dead cells in any group. Western Blot analysis showed no statistical difference in BAX or Bcl-2 expression between green OLED-treated and NT groups of hADSCs (Fig. 3c). Overall, these results indicated that the irradiation did not elevate apoptotic activities in 24 h green OLED-treated group.

### Upregulation of hADSC proliferation induced by green OLED irradiation

**Fig. 4:**
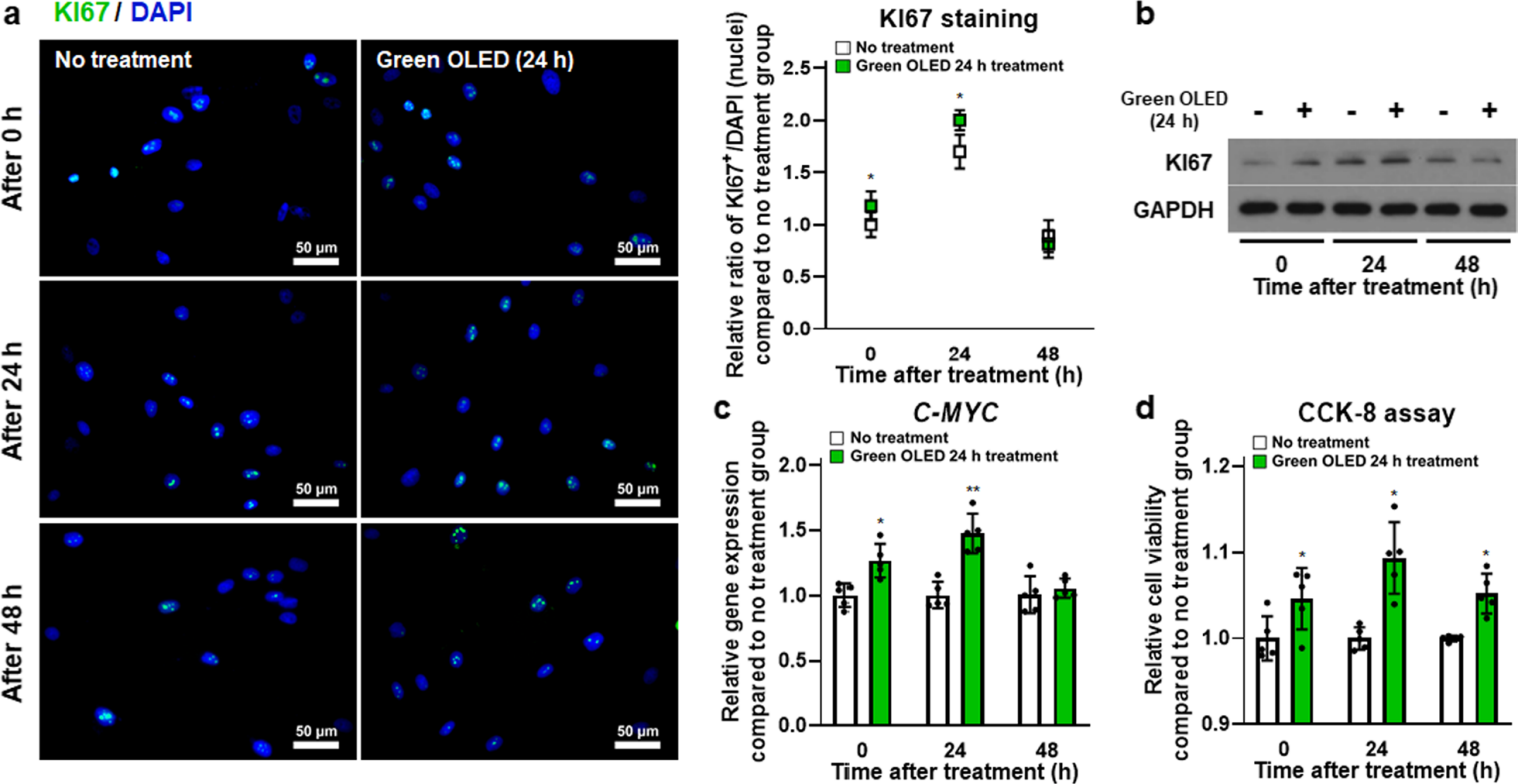
Increased proliferation of green OLED-treated hADSCs. **a** KI67 staining (blue: nuclei, green: KI67, scale bar = 50 µm) of hADSCs at 0, 24, and 48 h after green OLED irradiation for 24 h with quantification results (*n* = 6). **b** Western Blot analysis of KI67 expression in hADSCs at 0, 24, and 48 h after green OLED irradiation for 24 h. **c** Gene expression levels of *C-MYC* in hADSCs at 0, 24, and 48 h after green OLED irradiation for 24 h as evaluated by qRT-PCR (*n* = 5). **d** Cell proliferation of hADSCs at 0, 24, and 48 h after green OLED irradiation for 24 h as measured by CCK-8 assay (*n* = 5). *p < 0.05; **p < 0.001 compared to no treatment group with unpaired *t* test

As a cellular marker, KI67 was investigated to determine cell proliferation of hADSCs. Intracellular KI67 protein expression was analyzed by two kinds of assays. First, KI67 proteins were directly detected through immunocytochemistry (Fig. 4a). KI67 protein expression levels in both groups were increased at 24 h but decreased at 48 h. Meanwhile, there were significantly more KI67 proteins in treated hADSCs at 0 and 24 h post-irradiation than in untreated cells. Protein expression was analyzed qualitatively using Western Blot. Results showed the same tendency to KI67 staining (Fig. 4b). Moreover, *C-MYC* gene expression levels in green OLED-treated hADSCs were higher than in untreated controls with statistically significant differences at 0 and 24 h post-irradiation as assessed by qRT-PCR (Fig. 4c). Furthermore, the CCK-8 assay showed increased cell proliferation in green OLED-treated groups compared to each control (Fig. 4d). These data indicate that green OLED irradiation for 24 h can enhance the proliferation of hADSCs for at least 24 h after irradiation.

### Enhanced hADSC migration induced by green OLED irradiation

**Fig. 5:**
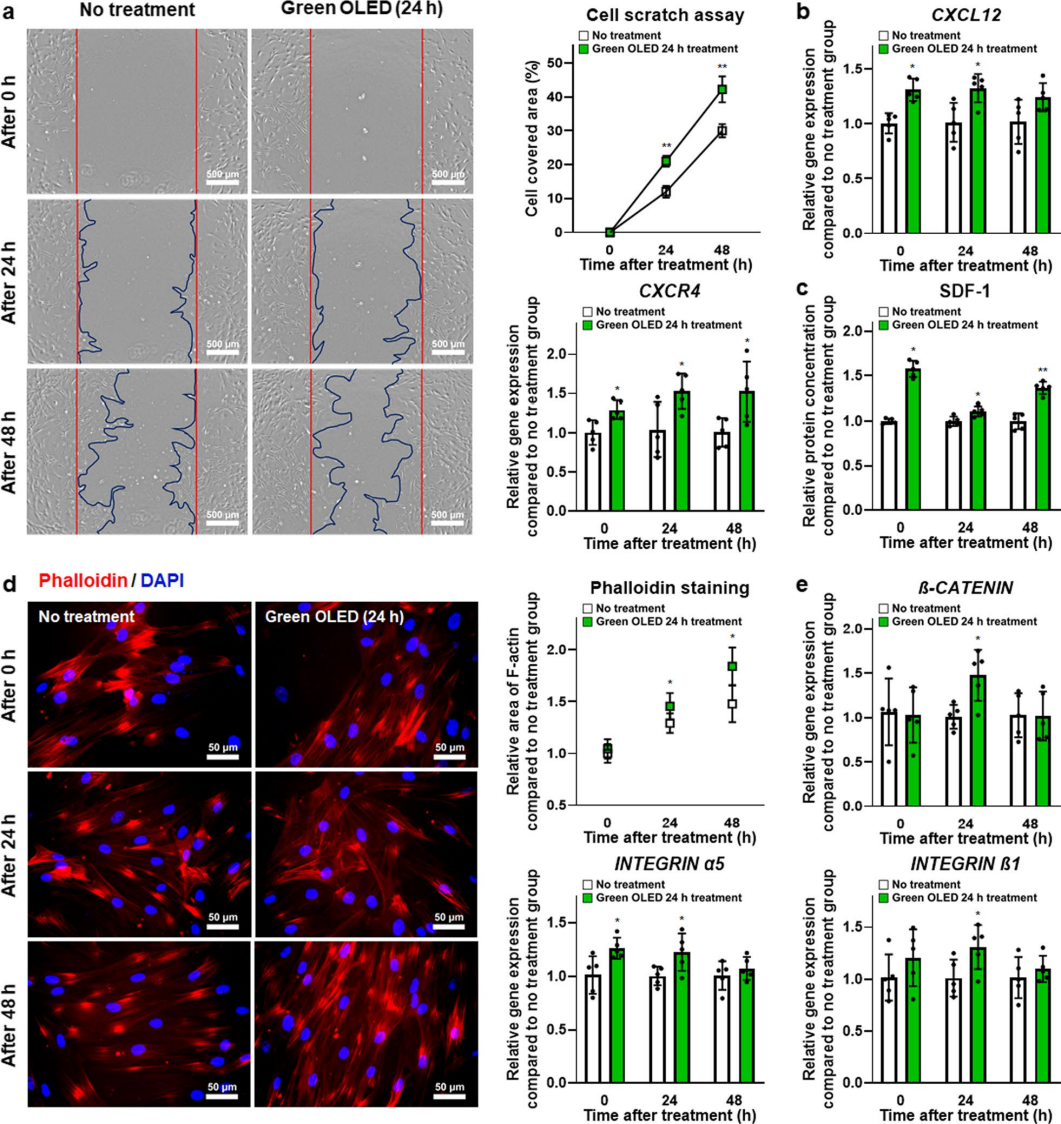
Enhanced migration and adhesion of green OLED-treated hADSCs. **a** Cell migration of hADSCs at 0, 24, and 48 h after green OLED irradiation for 24 h as evaluated by cell scratch assay (scale bar = 500 µm) with quantification results (*n* = 7). **b** Gene expression levels of *CXCL12* and *CXCR4* in hADSCs at 0, 24, and 48 h after green OLED irradiation for 24 h as evaluated by qRT-PCR (*n* = 5). **c** Secreted SDF-1 protein levels of hADSCs at 0, 24, and 48 h after green OLED irradiation for 24 h as evaluated by ELISA (*n* = 5). **d** Phalloidin staining (blue: nuclei, red: F-actin, scale bar = 50 µm) of hADSCs at 0, 24, and 48 h after green OLED irradiation for 24 h with quantification results (*n* = 6). **e** Gene expression levels of *β-CATENIN, INTEGRIN α5*, and *INTEGRIN β1* in hADSCs at 0, 24, and 48 h after green OLED irradiation for 24 h as evaluated by qRT-PCR (*n* = 5). *p < 0.05; **p < 0.001 compared to no treatment group with unpaired *t* test

Cell motility was examined by cell scratch assay. As shown in Fig. 5a, cell migration morphology was observed at 0, 24, and 48 h after green OLED irradiation. Covered area by treated cells was significantly larger than that of untreated control at 24 and 48 h after irradiation. Additionally, *CXCL12* and *CXCR4* gene expression levels in hADSCs were evaluated by qRT-PCR (Fig. 5b). Their gene expression levels in treated hADSCs were significantly higher than those in the NT group at every time point except for *CXCL12* at 48 h post-irradiation. Green OLED-treated hADSCs also secreted a significantly higher amount of SDF-1 than untreated cells at every time point (Fig. 5c). These data indicate that green OLED irradiation for 24 h could boost migration of hADSCs for approximately 48 h after irradiation.

### Enhancement of cell adhesion by green OLED irradiation

Phalloidin staining was performed to detect F-actin related to cell adhesion. Fluorescent stained hADSCs and F-actin were observed at 0, 24, and 48 h after green OLED irradiation (Fig. 5d). F-actin in the treated group occupied a significantly larger area than that in the NT group at 24 and 48 h after irradiation. Furthermore, gene expression levels of *β-CATENIN* at 24 h, *INTEGRIN α5* at 0 and 24 h, and *INTEGRIN β1* at 24 h post-green OLED irradiation were significantly upregulated in treated hADSCs (Fig. 5e). These data indicate that green OLED irradiation can improve adhesion of hADSCs for approximately 24 h after the irradiation.

### Effects of paracrine factors secreted from green OLED-treated hADSCs on cell proliferation

**Fig. 6:**
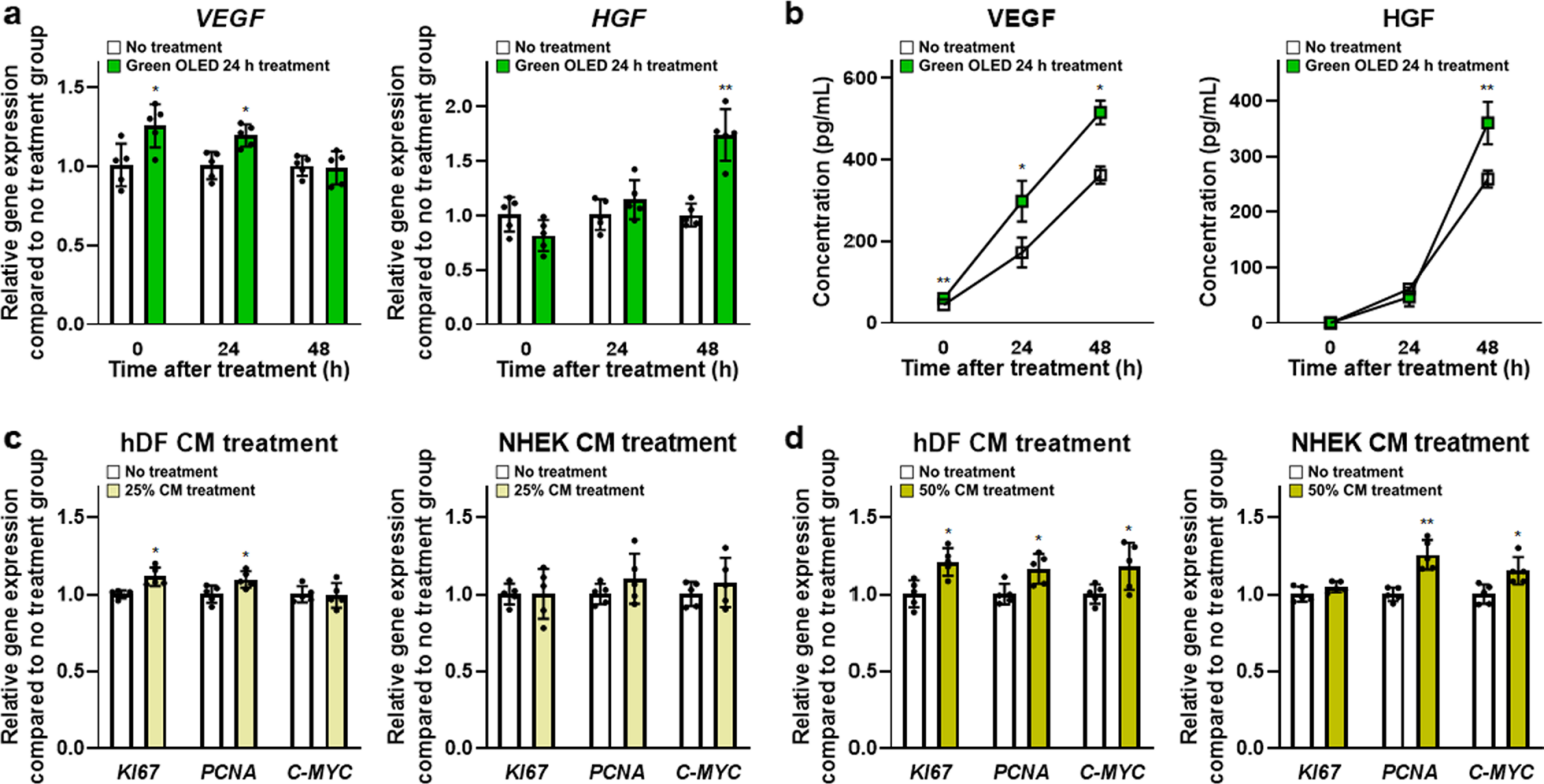
Enhanced secretion of growth factors in green OLED-treated hADSCs and proliferative effects of harvested CM. **a** Gene expression levels of *VEGF* and *HGF* in hADSCs at 0, 24, and 48 h after green OLED irradiation for 24 h as evaluated by qRT-PCR (*n* = 5). **b** VEGF and HGF proteins secreted from hADSCs at 0, 24, and 48 h after green OLED irradiation for 24 h as evaluated by ELISA (*n* = 5). Gene expression levels of *KI67, PCNA,* and *C-MYC* in hDFs and NHEKs cultured in 25% (**c**) and 50% (**d**) conditioned media as evaluated by qRT-PCR (*n* = 5). *p < 0.05; **p < 0.001 compared to no treatment group with unpaired *t* test

VEGF and HGF are known to play important roles in wound healing. In this study, *VEGF* and *HGF* gene expression levels in hADSCs were evaluated by qRT-PCR (Fig. 6a). Expression levels of *VEGF* at 0 and 24 h and *HGF* at 48 h post-green OLED irradiation were significantly increased compared to those in the NT group. However, they showed no significant difference at other time points. VEGF and HGF proteins secreted from hADSCs were detected using ELISA (Fig. 6b). This result was expected based on gene expression data. Compared to the NT group, green OLED-treated group showed significantly higher VEGF concentration at every time point. However, HGF concentration in the treated group was only higher at 48 h post-irradiation.

Therefore, conditioned media retrieved at 48 h after green OLED irradiation were used to demonstrate effects of paracrine factors on cell proliferation (Fig. 6c, d). Expression levels of *KI67*, *PCNA*, and *C-MYC* genes in hDFs treated with 25% and 50% CM for 48 h, but not *C-MYC* in 25%-CM-treated hDFs, were significantly upregulated than those in the NT group. In case of NHEKs, 50%-CM-treated cells had significantly enhanced gene expression levels of *PCNA* and *C-MYC* compared to control cells.

### Improved cutaneous wound healing by injecting green OLED-treated hADSCs

**Fig. 7:**
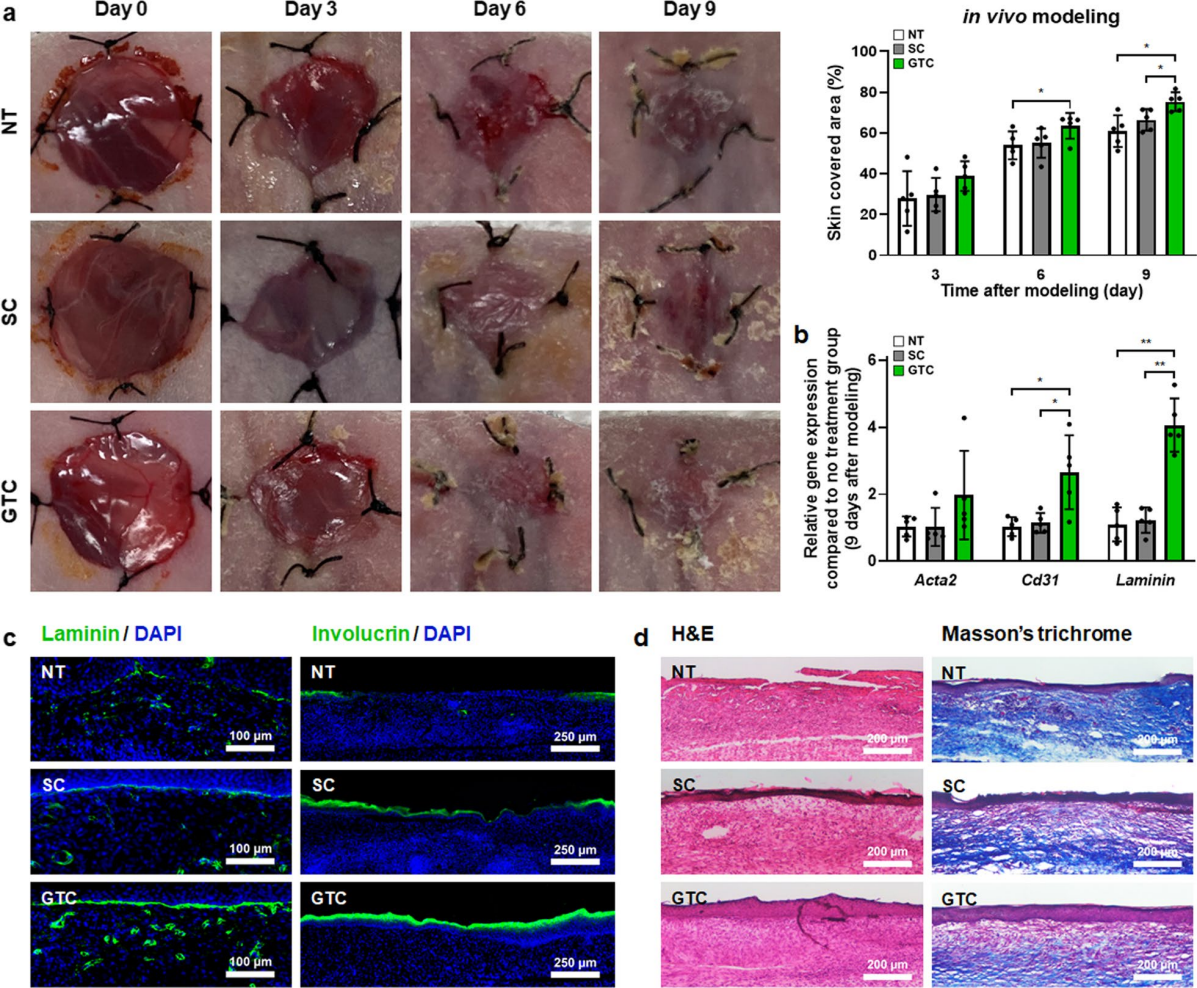
Improved in vivo wound healing in green OLED-treated hADSCs. **a** Representative photographs of skin wounds at 0, 3, 6, and 9 days after skin wound modeling and various treatments with wound coverage ratio quantification (*n* = 5). Mice were divided into three groups: no treatment (NT) served as control, hADSCs (SC), and green OLED-treated hADSCs (GTC) groups. **b** Gene expression levels of *Acta2, Cd31*, and *Laminin* in skin wound regions on day 9 as evaluated by qRT-PCR (*n* = 5). **c** Laminin (blue: nuclei, green: laminin, scale bar = 100 µm) and involucrin staining (blue: nuclei, green: involucrin, scale bar = 250 µm) of vertical sections of skin wound regions of each group on day 9. **d** H&E (scale bar = 200 µm) and Masson’s trichrome staining (scale bar = 200 µm) of vertical sections of skin wound regions of each group on day 9. *p < 0.05; **p < 0.001 compared to other groups with unpaired *t* test

According to previous experimental results, hADSCs harvested at 24 h after green OLED irradiation for 24 h were injected into mouse skin wound sites to promote tissue repair. As shown in Fig. 7a, the wound healing rate of the GTC group began to take the lead on day 6. The GTC group showed significantly higher wound healing rate than the NT group on day 6 and other groups on day 9. Moreover, gene expression levels of *Cd31* and *Laminin* in the GTC group were significantly increased compared to those in other groups (Fig. 7b).

Furthermore, the GTC group showed higher levels of laminin and involucrin protein signals than other groups based on immunohistochemistry (Fig. 7c). Additionally, histological images (Fig. 7d) of the GTC group revealed not only improved tissue regeneration, but also reduced inflammation and fibrosis through H&E and Masson’s trichrome staining.

## Discussion

By employing various techniques for photo-physical stimulation, PBM has been widely used to study the differentiation of stem cells or to enhance cellular functions such as proliferation and migration [42, 43]. Although various research studies on PBM have led to advancements overcoming limitations of stem cells therapy, more studies investigating detailed underlying molecular mechanisms of PBM are required. Technical challenges in application of PBM remained to be addressed. Previous studies have focused on the evaluation of cellular responses based on wavelength variance or tried to determine cellular responses caused by diverse conditions with an identical type of light source [44–46]. Notably, contrasting results have been observed when different types of cells are irradiated with light of the same wavelength [47, 48]. These studies show that the intracellular mechanisms triggered by PBM rely on the light source of PBM and the type of irradiated cells. In the present study, we used OLED light with a wavelength peak of 532 nm to irradiate hADSCs. We designed experiments to reveal biological changes in hADSCs promoted by green OLED irradiation and their underlying mechanisms. It has been suggested that CCO plays a major role in cellular responses of stem cell to PBM [49]. OPNs besides CCO are also related to responses to PBM [18]. Based on these reports, we examined changes in the expression of OPN family (OPN3 and OPN4) in hADSCs after green light-based PBM. Both *OPN3* and *OPN4* gene expression levels were upregulated after 24 h of irradiation. However, only OPN4 was increased at the protein level. Although further examination using various cell types is needed, these results may indicate that OPN4 is more sensitive to OLED light with 532 nm wavelength than OPN3 in hADSCs. Further supporting evidence of stimulation of OPN4 was gathered by determining ROS levels in hADSCs after OLED irradiation. Conigrave and Ward have reported that OPN4 can activate G_q_ protein, thereby increasing cytoplasmic Ca^2+^ concentration and ROS levels [22, 23]. Although we did not observe significant difference in intracellular Ca^2+^ concentration, both cellular ROS assay and H2DCFDA staining showed increases in ROS level and gene expression levels of *PLC-β* and *IP3R* known to mediate ER Ca^2+^ release [50] were elevated in green OLED-treated group of hADSCs. These results indicate that green OLED irradiation does not follow the molecular mechanism of TRP channels or the CCO pathway, the proposed mechanisms in PBM when using red or near-infrared light in the range of 600–700 nm and 780–1100 nm [49].

As shown in our results, green OLED treatment had no obvious cytotoxic effect on hADSCs despite generation of ROS. These results demonstrate that green OLED irradiation does not induce overexpression of ROS that can increase the cytotoxicity [51]. It has been reported that moderate level of ROS generation is associated with upregulation of growth factors such as VEGF which affects cell proliferation, migration, and adhesion of mesenchymal stem cells [52–54]. Accordingly, proliferation markers KI67 and *C-MYC* [55, 56] were increased at 24 h after OLED irradiation. CCK-8 assay result also showed that cell proliferation was enhanced with green OLED treatment. The reduction of KI67 and *C-MYC* expression at 48 h after irradiation demonstrates that our PBM-based approach has a 24 h validity. This may be lengthened with a longer irradiation time of high-intensity light. However, the possible cytotoxicity should be carefully analyzed. We observed that the increase in the motility of hADSCs was due to increased expression levels of *CXCL12*, *CXCR4*, and SDF-1 [57–59]. Increased level of *β-CATENIN*, *INTEGRIN α5*, and *INTEGRIN β1* after light irradiation is known to be associated with cell adhesion [60, 61], including adhesion of hADSCs. VEGF and HGF are widely known to play important roles in wound healing [62]. They were also upregulated in hADSCs irradiated with green OLED. Notably, CM obtained from irradiated hADSCs enhanced the expression of *KI67*, *PCNA* [63], and *C-MYC* genes in hDFs and NHEKs possibly due to increased amounts of VEGF and HGF in the CM of irradiated hADSCs. Here, hDFs and NHEKs are widely used cell types for in vitro assessment of wound healing [64]. For animal experiment, mouse cutaneous wound model was used. We observed the most prominent wound healing in the GTC group. Expression levels of *Cd31* gene (angiogenesis) [65] and *Laminin* gene (skin regeneration) [66] were increased at wound sites of mice injected with green OLED-treated hADSCs compared to those of mice in other groups. Immunohistochemical staining of laminin and involucrin [67] known to be associated with skin tissue regeneration showed significant increases in GTC-injected mice. Overall, our results verified that proliferation, migration, and adhesion of hADSCs as well as paracrine factor expression, all of which known to contribute to wound healing, were significantly enhanced after green OLED irradiation. Our approach suggests that green OLED-based PBM might serve as a novel preconditioning strategy to augment the wound healing efficacy of hADSCs.

## Conclusions

The present study demonstrates that a long-term mild PBM has better therapeutic efficacy than conventional methods. Parameters to maximize its efficacy are also suggested. We newly established photoreceptor molecular mechanism to hADSCs corresponding to green OLED used in this study. Importantly, cell proliferation, migration, adhesion, and paracrine factor expression in hADSCs are comprehensively enhanced by green OLED irradiation. These effects lasted for 24–48 h. These results indicate that green OLED can be utilized for cell culture without worrying about differentiation or mutation of cells. Furthermore, preclinical values of green OLED-treated hADSCs were verified with a mouse wound model compared to conventional cell therapy. Collectively, this report provides a concept of wound healing improved by hADSCs, including OPN4 stimulated by OLED irradiation with a wavelength peak of 532 nm.

## Data Availability

The data generated and/or analyzed during this study are available from the corresponding authors on reasonable request.

## Abbreviations

ATP: Adenosine triphosphate
Ca^2+^: Calcium ions
CCO: Cytochrome c oxidase
CM: Conditioned medium
ER: Endoplasmic reticulum
GPCR: G-protein-coupled receptor
GTC: Green OLED-treated human adipose-derived stem cell
hADSC: Human adipose-derived stem cell
NT: No treatment
OLED: Organic light-emitting diode
OPN: Opsin
PBM: Photobiomodulation
ROS: Reactive oxygen species
TRP: Transient receptor potential

## References

[1] Tsuji W, Rubin JP, Marra KG (2014). Adipose-derived stem cells: implications in tissue regeneration. World J Stem Cells.

[2] Hong SJ, Traktuev DO, March KL (2010). Therapeutic potential of adipose-derived stem cells in vascular growth and tissue repair. Curr Opin Organ Transplant.

[3] Gentile P, Sterodimas A, Calabrese C, Garcovich S (2021). Systematic review: advances of fat tissue engineering as bioactive scaffold, bioactive material, and source for adipose-derived mesenchymal stem cells in wound and scar treatment. Stem Cell Res Ther.

[4] Gentile P, Sterodimas A, Pizzicannella J, Calabrese C, Garcovich S (2020). Research progress on mesenchymal stem cells (MSCs), adipose-derived mesenchymal stem cells (AD-MSCs), drugs, and vaccines in inhibiting COVID-19 disease. Aging Dis.

[5] Mazini L, Rochette L, Admou B, Amal S, Malka G (2020). Hopes and limits of adipose-derived stem cells (ADSCs) and mesenchymal stem cells (MSCs) in wound healing. Int J Mol Sci.

[6] Ong WK, Chakraborty S, Sugii S (2021). Adipose tissue: understanding the heterogeneity of stem cells for regenerative medicine. Biomolecules.

[7] Zhu M, Kohan E, Bradley J, Hedrick M, Benhaim P, Zuk P (2009). The effect of age on osteogenic, adipogenic and proliferative potential of female adipose-derived stem cells. J Tissue Eng Regen Med.

[8] Beugels J, Molin DG, Ophelders DR, Rutten T, Kessels L, Kloosterboer N (2019). Electrical stimulation promotes the angiogenic potential of adipose-derived stem cells. Sci Rep.

[9] Gentile P, Garcovich S (2021). Systematic review: adipose-derived mesenchymal stem cells, platelet-rich plasma and biomaterials as new regenerative strategies in chronic skin wounds and soft tissue defects. Int J Mol Sci.

[10] Corazza AV, Jorge J, Kurachi C, Bagnato VS (2007). Photobiomodulation on the angiogenesis of skin wounds in rats using different light sources. Photomed Laser Surg.

[11] Gentile P, Garcovich S, Lee S-I, Han S (2021). Regenerative biotechnologies in plastic surgery: a multicentric, retrospective, case-series study on the use of micro-needling with low-level light/laser therapy as a hair growth boost in patients affected by androgenetic alopecia. Appl Sci.

[12] Ginani F, Soares DM, Barboza CAG (2015). Effect of low-level laser therapy on mesenchymal stem cell proliferation: a systematic review. Lasers Med Sci.

[13] Hamblin MR (2017). Mechanisms and applications of the anti-inflammatory effects of photobiomodulation. AIMS Biophys.

[14] Farivar S, Malekshahabi T, Shiari R (2014). Biological effects of low level laser therapy. J Lasers Med Sci.

[15] Sazanov LA (2015). A giant molecular proton pump: structure and mechanism of respiratory complex I. Nat Rev Mol Cell Biol.

[16] Michel H, Behr J, Harrenga A, Kannt A (1998). Cytochrome c oxidase: structure and spectroscopy. Annu Rev Biophys Biomol Struct.

[17] Srinivasan S, Avadhani NG (2012). Cytochrome c oxidase dysfunction in oxidative stress. Free Radic Biol Med.

[18] Suh S, Choi EH, Atanaskova MN (2020). The expression of opsins in the human skin and its implications for photobiomodulation: a systematic review. Photodermatol Photoimmunol Photomed.

[19] Castellano-Pellicena I, Uzunbajakava NE, Mignon C, Raafs B, Botchkarev VA, Thornton MJ (2019). Does blue light restore human epidermal barrier function via activation of Opsin during cutaneous wound healing?. Lasers Surg Med.

[20] Serrage H, Heiskanen V, Palin WM, Cooper PR, Milward MR, Hadis M (2019). Under the spotlight: mechanisms of photobiomodulation concentrating on blue and green light. Photochem Photobiol Sci.

[21] Ramsey IS, Delling M, Clapham DE (2006). An introduction to TRP channels. Annu Rev Physiol.

[22] Conigrave AD, Ward DT (2013). Calcium-sensing receptor (CaSR): pharmacological properties and signaling pathways. Best Pract Res Clin Endocrinol Metab.

[23] Gordeeva A, Zvyagilskaya R, Labas YA (2003). Cross-talk between reactive oxygen species and calcium in living cells. Biochem (Mosc).

[24] Mvula B, Mathope T, Moore T, Abrahamse H (2008). The effect of low level laser irradiation on adult human adipose derived stem cells. Lasers Med Sci.

[25] Hamblin MR (2018). Mechanisms and mitochondrial redox signaling in photobiomodulation. Photochem Photobiol.

[26] Joensen J, Demmink JH, Johnson MI, Iversen VV, Lopes-Martins RÁB, Bjordal JM (2011). The thermal effects of therapeutic lasers with 810 and 904 nm wavelengths on human skin. Photomed Laser Surg.

[27] Wang Y, Huang Y-Y, Wang Y, Lyu P, Hamblin MR (2017). Red (660 nm) or near-infrared (810 nm) photobiomodulation stimulates, while blue (415 nm), green (540 nm) light inhibits proliferation in human adipose-derived stem cells. Sci Rep.

[28] Lev-Tov H, Mamalis A, Brody N, Siegel D, Jagdeo J (2013). Inhibition of fibroblast proliferation in vitro using red light-emitting diodes. Dermatol Surg.

[29] Wang Y, Huang Y-Y, Wang Y, Lyu P, Hamblin MR (2016). Photobiomodulation (blue and green light) encourages osteoblastic-differentiation of human adipose-derived stem cells: role of intracellular calcium and light-gated ion channels. Sci Rep.

[30] Amid R, Kadkhodazadeh M, Ahsaie MG, Hakakzadeh A (2014). Effect of low level laser therapy on proliferation and differentiation of the cells contributing in bone regeneration. J Lasers Med Sci.

[31] Ahrabi B, Tavirani MR, Khoramgah MS, Noroozian M, Darabi S, Khoshsirat S (2019). The effect of photobiomodulation therapy on the differentiation, proliferation, and migration of the mesenchymal stem cell: a review. J Lasers Med Sci.

[32] Hendudari F, Piryaei A, Hassani S-N, Darbandi H, Bayat M (2016). Combined effects of low-level laser therapy and human bone marrow mesenchymal stem cell conditioned medium on viability of human dermal fibroblasts cultured in a high-glucose medium. Lasers Med Sci.

[33] Fushimi T, Inui S, Nakajima T, Ogasawara M, Hosokawa K, Itami S (2012). Green light emitting diodes accelerate wound healing: characterization of the effect and its molecular basis in vitro and in vivo. Wound Repair Regen.

[34] Anwer AG, Gosnell ME, Perinchery SM, Inglis DW, Goldys EM (2012). Visible 532 nm laser irradiation of human adipose tissue-derived stem cells: effect on proliferation rates, mitochondria membrane potential and autofluorescence. Lasers Surg Med.

[35] Hassan WU, Greiser U, Wang W (2014). Role of adipose-derived stem cells in wound healing. Wound Repair Regen.

[36] Gentile P, Garcovich S (2019). Concise review: adipose-derived stem cells (ASCs) and adipocyte-secreted exosomal microRNA (A-SE-miR) modulate cancer growth and promote wound repair. J Clin Med.

[37] Wang FW, Wang Z, Zhang YM, Du ZX, Zhang XL, Liu Q (2013). Protective effect of melatonin on bone marrow mesenchymal stem cells against hydrogen peroxide-induced apoptosis in vitro. J Cell Biochem.

[38] Liang C-C, Park AY, Guan J-L (2007). In vitro scratch assay: a convenient and inexpensive method for analysis of cell migration in vitro. Nat Protoc.

[39] Bobadilla AVP, Arévalo J, Sarró E, Byrne HM, Maini PK, Carraro T (2019). In vitro cell migration quantification method for scratch assays. J R Soc Interface.

[40] Wang X, Ge J, Tredget EE, Wu Y (2013). The mouse excisional wound splinting model, including applications for stem cell transplantation. Nat Protoc.

[41] Kruse CR, Nuutila K, Lee CC, Kiwanuka E, Singh M, Caterson EJ (2015). The external microenvironment of healing skin wounds. Wound Repair Regen.

[42] de Andrade ALM, Luna GF, Brassolatti P, Leite MN, Parisi JR, de Oliveira Leal ÂM (2019). Photobiomodulation effect on the proliferation of adipose tissue mesenchymal stem cells. Lasers Med Sci.

[43] Zamani ARN, Saberianpour S, Geranmayeh MH, Bani F, Haghighi L, Rahbarghazi R (2020). Modulatory effect of photobiomodulation on stem cell epigenetic memory: a highlight on differentiation capacity. Lasers Med Sci.

[44] Pinto H, Oliver PG, Mengual ES-V (2021). The effect of photobiomodulation on human mesenchymal cells: a literature review. Aesthet Plast Surg.

[45] Rohringer S, Holnthoner W, Chaudary S, Slezak P, Priglinger E, Strassl M (2017). The impact of wavelengths of LED light-therapy on endothelial cells. Sci Rep.

[46] Tani A, Chellini F, Giannelli M, Nosi D, Zecchi-Orlandini S, Sassoli C (2018). Red (635 nm), near-infrared (808 nm) and violet-blue (405 nm) photobiomodulation potentiality on human osteoblasts and mesenchymal stromal cells: a morphological and molecular in vitro study. Int J Mol Sci.

[47] AlGhamdi KM, Kumar A, Moussa NA (2012). Low-level laser therapy: a useful technique for enhancing the proliferation of various cultured cells. Lasers Med Sci.

[48] Fekrazad R, Asefi S, Eslaminejad MB, Taghiar L, Bordbar S, Hamblin MR (2019). Photobiomodulation with single and combination laser wavelengths on bone marrow mesenchymal stem cells: proliferation and differentiation to bone or cartilage. Lasers Med Sci.

[49] de Freitas LF, Hamblin MR (2016). Proposed mechanisms of photobiomodulation or low-level light therapy. IEEE J Sel Top Quantum Electron.

[50] Patergnani S, Suski JM, Agnoletto C, Bononi A, Bonora M, De Marchi E (2011). Calcium signaling around mitochondria associated membranes (MAMs). Cell Commun Signal.

[51] Carrasco-Torres G, Baltiérrez-Hoyos R, Andrade-Jorge E, Villa-Treviño S, Trujillo-Ferrara JG, Vásquez-Garzón VR (2017). Cytotoxicity, oxidative stress, cell cycle arrest, and mitochondrial apoptosis after combined treatment of hepatocarcinoma cells with maleic anhydride derivatives and quercetin. Oxid Med Cell Longev.

[52] Sart S, Song L, Li Y (2015). Controlling redox status for stem cell survival, expansion, and differentiation. Oxid Med Cell Longev.

[53] Nugud A, Sandeep D, El-Serafi AT (2018). Two faces of the coin: minireview for dissecting the role of reactive oxygen species in stem cell potency and lineage commitment. J Adv Res.

[54] Hurd TR, DeGennaro M, Lehmann R (2012). Redox regulation of cell migration and adhesion. Trends Cell Biol.

[55] Kakudo N, Morimoto N, Ma Y, Kusumoto K (2019). Differences between the proliferative effects of human platelet lysate and fetal bovine serum on human adipose-derived stem cells. Cells.

[56] Paula AC, Martins TM, Zonari A, Frade SP, Angelo PC, Gomes DA (2015). Human adipose tissue-derived stem cells cultured in xeno-free culture condition enhance c-MYC expression increasing proliferation but bypassing spontaneous cell transformation. Stem Cell Res Ther.

[57] Zhang L, Zhou Y, Sun X, Zhou J, Yang P (2017). CXCL12 overexpression promotes the angiogenesis potential of periodontal ligament stem cells. Sci Rep.

[58] Bobis-Wozowicz S, Miekus K, Wybieralska E, Jarocha D, Zawisz A, Madeja Z (2011). Genetically modified adipose tissue-derived mesenchymal stem cells overexpressing CXCR4 display increased motility, invasiveness, and homing to bone marrow of NOD/SCID mice. Exp Hematol.

[59] Li Q, Guo Y, Chen F, Liu J, Jin P (2016). Stromal cell-derived factor-1 promotes human adipose tissue-derived stem cell survival and chronic wound healing. Exp Ther Med.

[60] Yan W, Lin C, Guo Y, Chen Y, Du Y, Lau WB (2020). N-cadherin overexpression mobilizes the protective effects of mesenchymal stromal cells against ischemic heart injury through a β-catenin-dependent manner. Circ Res.

[61] Albelda SM, Buck CA (1990). Integrins and other cell adhesion molecules. FASEB J.

[62] Nie C, Yang D, Xu J, Si Z, Jin X, Zhang J (2011). Locally administered adipose-derived stem cells accelerate wound healing through differentiation and vasculogenesis. Cell Transplant.

[63] Wu W-S, Wang F-S, Yang KD, Huang C-C, Kuo Y-R (2006). Dexamethasone induction of keloid regression through effective suppression of VEGF expression and keloid fibroblast proliferation. J Investig Dermatol.

[64] Duan-Arnold Y, Gyurdieva A, Johnson A, Jacobstein DA, Danilkovitch A (2015). Soluble factors released by endogenous viable cells enhance the antioxidant and chemoattractive activities of cryopreserved amniotic membrane. Adv Wound Care.

[65] Mildner M, Hacker S, Haider T, Gschwandtner M, Werba G, Barresi C (2013). Secretome of peripheral blood mononuclear cells enhances wound healing. PLoS ONE.

[66] Senyürek I, Kempf WE, Klein G, Maurer A, Kalbacher H, Schäfer L (2014). Processing of laminin α chains generates peptides involved in wound healing and host defense. J Innate Immun.

[67] Yang DH, Seo DI, Lee D-W, Bhang SH, Park K, Jang G (2017). Preparation and evaluation of visible-light cured glycol chitosan hydrogel dressing containing dual growth factors for accelerated wound healing. J Ind Eng Chem.

